# Inequalities and asymptotic expansions related to the generalized Somos quadratic recurrence constant

**DOI:** 10.1186/s13660-018-1741-8

**Published:** 2018-06-27

**Authors:** Xue-Si Ma, Chao-Ping Chen

**Affiliations:** School of Mathematics and Informatics, Henan Polytechnic University, Jiaozuo City, China

**Keywords:** 41A60, 26D15, Somos’ quadratic recurrence constant, Inequality, Asymptotic expansion, Generalized Euler constant

## Abstract

In this paper, we give asymptotic expansions and inequalities related to the generalized Somos quadratic recurrence constant, using its relation with the generalized Euler constant.

## Introduction

Somos’ quadratic recurrence constant is defined (see [1–3]) by

1.1 $$ \begin{aligned}[b] \sigma &=\sqrt{1\sqrt{2\sqrt{3\cdots }}}=\prod _{n=1}^{\infty }n ^{1/2^{n}}=\prod _{k=1}^{\infty } \biggl( 1+\frac{1}{k} \biggr) ^{1/2^{k}}= \exp \Biggl\{ \sum_{k=1}^{\infty } \frac{\ln k}{2^{k}} \Biggr\} \\ & =1.66168794 \ldots\end{aligned} $$

or

1.2 $$ \sigma =\exp \biggl\{ - \int_{0}^{1}\frac{1-x}{(2-x)\ln x}\,\mathrm{d}x \biggr\} = \exp \biggl\{ - \int_{0}^{1} \int_{0}^{1}\frac{x}{(2-xy)\ln (xy)} \,\mathrm{d}x \, \mathrm{d}y \biggr\} . $$

The constant *σ* arises in the study of the asymptotic behavior of the sequence

1.3 $$ g_{0}=1,\quad\quad g_{n}=ng_{n-1}^{2}, \quad n \in \mathbb{N}:=\{1,2,3,\ldots \}, $$

with the first few terms

$$ g_{0}=1,\quad\quad g_{1}=1,\quad\quad g_{2}=2, \quad\quad g_{3}=12,\quad\quad g_{4}=576,\quad\quad g_{5}=1{,}658{,}880,\quad\quad \ldots . $$

This sequence behaves as follows (see [4, p. 446] and [3, 5]):

1.4 $$\begin{aligned} \begin{aligned}[b] g_{n} &\sim \frac{\sigma^{2^{n}}}{n} \biggl(1+ \frac{2}{n}- \frac{1}{n^{2}}+\frac{4}{n^{3}}-\frac{21}{n^{4}}+ \frac{138}{n^{5}}-\frac{1091}{n ^{6}}+\frac{10{,}088}{n^{7}} -\frac{106{,}918}{n^{8}} \\ & \quad{} +\frac{1{,}279{,}220}{n^{9}}-\frac{17{,}070{,}418}{n^{10}}+ \frac{251{,}560{,}472}{n^{11}}- \frac{4{,}059{,}954{,}946}{n^{12}}+\cdots \biggr)^{-1}. \end{aligned} \end{aligned}$$

The constant *σ* appears in important problems from pure and applied analysis, and it is the motivation for a large number of research papers (see, for example, [1, 6–16]).

Sondow and Hadjicostas [15] introduced and studied the generalized-Euler-constant function $\gamma (z)$, defined by

1.5 $$\begin{aligned} \gamma (z)=\sum_{n=1}^{\infty }z^{n-1} \biggl( \frac{1}{n}-\ln \frac{n+1}{n} \biggr) , \end{aligned}$$

where the series converges when $\vert z \vert \leq 1$. Pilehrood and Pilehrood [13] considered the function $z\gamma (z)$ ($\vert z \vert \leq 1$). The function $\gamma (z)$ generalizes both Euler’s constant $\gamma (1)$ and the alternating Euler constant $\ln \frac{4}{\pi } = \gamma (-1)$ [17, 18].

Sondow and Hadjicostas [15] defined the generalized Somos constant

1.6 $$\begin{aligned} \sigma_{t}=\sqrt[t]{1\sqrt[t]{2\sqrt[t]{3\sqrt[t]{4 \cdots }}}}=\prod_{n=1}^{\infty }n^{1/t^{n}}= \biggl( \frac{t}{t-1} \biggr) ^{1/(t-1)} \exp \biggl\{ - \frac{1}{t(t-1)}\gamma \biggl( \frac{1}{t} \biggr) \biggr\} , \quad t>1. \end{aligned}$$

Coffey [19] gave the integral and series representations for $\ln \sigma_{t}$:

1.7 $$\begin{aligned} \ln \sigma_{t}= \int_{0}^{\infty } \biggl( \frac{e^{-x}}{t-1}+ \frac{1}{1-te ^{x}} \biggr) \frac{\mathrm{d}x}{x} \end{aligned}$$

and

1.8 $$\begin{aligned} \ln \sigma_{t}=\frac{1}{t-1}\sum _{k=1}^{\infty }\frac{(-1)^{k-1}}{k} \operatorname{Li}_{k} \biggl( \frac{1}{t} \biggr) =\frac{1}{t-1}\sum _{k=1}^{ \infty }\frac{1}{k} \biggl[ t \operatorname{Li}_{k} \biggl( \frac{1}{t} \biggr) -1 \biggr] \end{aligned}$$

in terms of the polylogarithm function.

It is known (see [15]) that

1.9 $$ \gamma \biggl( \frac{1}{2} \biggr) =2\ln \frac{2}{\sigma }, \quad \text{equivalently},\quad \sigma =2\exp \biggl\{ - \frac{1}{2}\gamma \biggl( \frac{1}{2} \biggr) \biggr\} . $$

Thus, formula (1.5) is closely related to Somos’ quadratic recurrence constant *σ*.

Define

$$\begin{aligned} \gamma_{n}(z)=\sum_{k=1}^{n}z^{k-1} \biggl( \frac{1}{k}-\ln \frac{k+1}{k} \biggr) , \quad \vert z \vert \leq 1. \end{aligned}$$

Mortici [11] proved that for $n\in \mathbb{N}$,

1.10 $$\begin{aligned} \frac{270(n +1)}{2^{n}(270n^{3} + 1530n^{2} + 1065n + 6293)}< \gamma \biggl( \frac{1}{2} \biggr) - \gamma_{n} \biggl( \frac{1}{2} \biggr) < \frac{18}{2^{n}(18n ^{2} +84n - 13)} \end{aligned}$$

and

1.11 $$\begin{aligned} &\sum_{k=n+1}^{\infty } \frac{1}{2k^{2}\cdot 3^{k-1}}- \frac{22{,}400(n+1)}{3^{n}(44{,}800n ^{4}+280{,}000n^{3}+435{,}120n^{2}+744{,}380n-2{,}477{,}677)} \\ &\quad < \gamma \biggl( \frac{1}{3} \biggr) -\gamma_{n} \biggl( \frac{1}{3} \biggr) < \sum_{k=n+1}^{\infty } \frac{1}{2k^{2}\cdot 3^{k-1}}-\frac{160}{3^{n}(320n ^{3} + 1680n^{2} + 1428n + 3889)}. \end{aligned}$$

Lu and Song [10] improved Mortici’s results and obtained the inequalities:

1.12 $$\begin{aligned} \begin{aligned}[b] \frac{690n^{2} + 3524n + 145}{6(2^{n})(n +1)^{2}(115n^{2} + 894n + 779)}&< \gamma \biggl( \frac{1}{2} \biggr) - \gamma_{n} \biggl( \frac{1}{2} \biggr) \\ &< \frac{48n + 127}{3(2^{n})(16n + 85)(n +1)^{2}}\end{aligned} \end{aligned}$$

and

1.13 $$\begin{aligned} &\sum_{k=n+1}^{\infty } \frac{1}{2k^{2}\cdot 3^{k-1}}- \frac{987 840n ^{2} + 8 444 340n + 10 946 779}{40(3^{n})(n +1)^{2}(49 392n^{3} + 582 741n^{2} + 1 769 516n + 1 236 167)} \\ &\quad < \gamma \biggl( \frac{1}{3} \biggr) -\gamma_{n} \biggl( \frac{1}{3} \biggr) < \sum_{k=n+1}^{\infty } \frac{1}{2k^{2}\cdot 3^{k-1}}-\frac{1620n^{2} + 6995n + 1847}{40(3^{n})(81n^{2} + 532n + 451)(n +1)^{3}} \end{aligned}$$

for $n\in \mathbb{N}$.

You and Chen [16] further improved inequalities (1.10)–(1.13). Recently, Chen and Han [7] gave new bounds for $\gamma (1/2)-\gamma_{n}(1/2)$:

1.14 $$\begin{aligned} &\frac{1}{2^{n}} \biggl( \frac{1}{(n+1)^{2}}- \frac{8}{3(n+1)^{3}}+ \frac{23}{2(n+1)^{4}}-\frac{332}{5(n+1)^{5}}+\frac{479}{(n+1)^{6}}- \frac{29{,}024}{7(n+1)^{7}} \biggr) \\ &\quad < \gamma \biggl( \frac{1}{2} \biggr) -\gamma_{n} \biggl( \frac{1}{2} \biggr) \\ &\quad < \frac{1}{2^{n}} \biggl( \frac{1}{(n+1)^{2}}- \frac{8}{3(n+1)^{3}}+ \frac{23}{2(n+1)^{4}}-\frac{332}{5(n+1)^{5}}+\frac{479}{(n+1)^{6}} \biggr) \end{aligned}$$

for $n\in \mathbb{N}$, and presented the following asymptotic expansion:

1.15 $$\begin{aligned} \begin{aligned}[b] &\gamma \biggl( \frac{1}{2} \biggr) - \gamma_{n} \biggl( \frac{1}{2} \biggr) \\ & \quad \sim \frac{1}{2^{n}(n+1)^{2}} \biggl\{ 1- \frac{8}{3(n+1)}+ \frac{23}{2(n+1)^{2}}-\frac{332}{5(n+1)^{3}}+\frac{479}{(n+1)^{4}}- \cdots \biggr\} \end{aligned} \end{aligned}$$

as $n\to \infty $. Moreover, these authors gave a formula for successively determining the coefficients in (1.15).

Chen and Han [7] pointed out that the lower bound in (1.14) is for $n\geq 24$ sharper than the one in (1.12), and the upper bound in (1.14) is for $n\geq 18$ sharper than the one in (1.12),

For any positive integer $m\geq 2$, in this paper we give the asymptotic expansion of $\gamma ( 1/m ) -\gamma_{n} ( 1/m ) $ as $n\to \infty $. Based on the result obtained, we establish the inequality for $\gamma ( 1/4 ) -\gamma_{n} ( 1/4 ) $. We also consider the asymptotic expansion for $\gamma ( -1 ) - \gamma_{n} ( -1 ) $.

## Lemmas

### Lemma 2.1

*As* $x\to \infty $,

2.1 $$ \frac{1}{x}-\ln \biggl( 1+\frac{1}{x} \biggr) -\sum _{j=2}^{m-1} \frac{(-1)^{j}}{j} \frac{1}{x^{j}}\sim A(x)-\frac{1}{m}A(x+1), $$

*where* $A(x)$ *is defined by*

2.2 $$ A(x)=\sum_{j=m}^{\infty } \frac{a_{j}}{x^{j}} $$

*with the coefficients* $a_{j}$ *given by the recurrence relation*

2.3 $$\begin{aligned} a_{j}=\frac{(-1)^{j}}{m-1} \Biggl\{ \frac{m}{j}+ \sum_{k=m}^{j-1}(-1)^{k}a _{k}\binom{j-1}{j-k} \Biggr\} , \quad j\geq m. \end{aligned}$$

*Here*, *and throughout this paper*, *an empty sum is understood to be zero*.

### Proof

Using the Maclaurin series of $\ln (1+t)$,

$$ \ln (1+t)=\sum_{j=1}^{\infty } \frac{(-1)^{j-1}}{j}t^{j}, \quad -1< t\leq 1, $$

we obtain

2.4 $$ \frac{1}{x}-\ln \biggl( 1+\frac{1}{x} \biggr) -\sum _{j=2}^{m-1} \frac{(-1)^{j}}{j} \frac{1}{x^{j}}=\sum_{j=m}^{\infty } \frac{(-1)^{j}}{j}\frac{1}{x^{j}}. $$

In view of (2.4), we can let

2.5 $$ \frac{1}{x}-\ln \biggl( 1+\frac{1}{x} \biggr) -\sum _{j=2}^{m-1} \frac{(-1)^{j}}{j} \frac{1}{x^{j}}\sim \sum_{j=m}^{\infty } \frac{a_{j}}{x ^{j}}-\frac{1}{m}\sum_{j=m}^{\infty } \frac{a_{j}}{(x+1)^{j}}, $$

where $a_{j}$ are real numbers to be determined.

Write (2.5) as

2.6 $$ \frac{1}{x}-\ln \biggl( 1+\frac{1}{x} \biggr) -\sum _{j=2}^{m-1} \frac{(-1)^{j}}{j} \frac{1}{x^{j}}\sim \sum_{j=m}^{\infty } \frac{a_{j}}{x ^{j}}-\frac{1}{m}\sum_{j=m}^{\infty } \frac{a_{j}}{x^{j}} \biggl( 1+ \frac{1}{x} \biggr) ^{-j}. $$

Direct computation yields

2.7 $$\begin{aligned} \sum_{j=m}^{\infty } \frac{a_{j}}{x^{j}} \biggl( 1+\frac{1}{x} \biggr) ^{-j} &=\sum_{j=m}^{\infty } \frac{a_{j}}{x^{j}}\sum_{k=0}^{\infty } \binom{-j}{k}\frac{1}{x^{k}} \\ &=\sum_{j=m}^{\infty }\frac{a_{j}}{x^{j}}\sum _{k=0}^{\infty }(-1)^{k} \binom{k+j-1}{k}\frac{1}{x^{k}} \\ &=\sum_{j=m}^{\infty }\sum _{k=m}^{j}a_{k}(-1)^{j-k} \binom{j-1}{j-k}\frac{1}{x ^{j}}. \end{aligned}$$

It follows from (2.4), (2.6), and (2.7) that

2.8 $$ \sum_{j=m}^{\infty } \frac{(-1)^{j}}{j}\frac{1}{x^{j}}\sim \sum_{j=m} ^{\infty } \Biggl\{ a_{j}-\frac{1}{m}\sum _{k=m}^{j}a_{k}(-1)^{j-k} \binom{j-1}{j-k} \Biggr\} \frac{1}{x^{j}}. $$

Equating coefficients of the term $x^{-j}$ on both sides of (2.8) yields

2.9 $$ \frac{(-1)^{j}}{j}=a_{j}-\frac{1}{m}\sum _{k=m}^{j}a_{k}(-1)^{j-k} \binom{j-1}{j-k}, \quad j\geq m. $$

For $j=m$, we obtain $a_{m}=\frac{(-1)^{m}}{m-1}$, and for $j\geq m+1$, we have

$$ \frac{(-1)^{j}}{j}=a_{j}-\frac{1}{m} \Biggl[ \sum _{k=m}^{j-1}a_{k}(-1)^{j-k} \binom{j-1}{j-k}+a_{j} \Biggr] , \quad j\geq m+1. $$

We then obtain the recursive formula

$$\begin{aligned} a_{m}=\frac{(-1)^{m}}{m-1}, \quad\quad a_{j}= \frac{(-1)^{j}m}{(m-1)j}+ \frac{1}{m-1}\sum_{k=m}^{j-1}a_{k}(-1)^{j-k} \binom{j-1}{j-k}, \quad j\geq m+1, \end{aligned}$$

which can be written as (2.3). The proof of Lemma 2.1 is complete. □

### Lemma 2.2

*Let*

2.10 $$ a(x)=\frac{1}{3x^{4}}-\frac{32}{45x^{5}}\quad \textit{and}\quad b(x)=\frac{1}{3x ^{4}}-\frac{32}{45x^{5}}+\frac{68}{27x^{6}}. $$

*Then*, *for* $x\geq 1$,

2.11 $$ a(x)-\frac{1}{4}a(x+1)< \frac{1}{x}-\ln \biggl( 1+ \frac{1}{x} \biggr) -\frac{1}{2x ^{2}}+\frac{1}{3x^{3}}< b(x)- \frac{1}{4}b(x+1). $$

### Proof

It is well known that for $-1< t\leq 1$ and $m\in \mathbb{N}$,

$$ \sum_{j=1}^{2m}\frac{(-1)^{j-1}}{j}t^{j}< \ln (1+t)< \sum_{j=1}^{2m-1} \frac{(-1)^{j-1}}{j}t^{j}, $$

which implies that for $x\geq 1$ and $m\geq 2$,

2.12 $$ \sum_{j=4}^{2m+1} \frac{(-1)^{j}}{jx^{j}}< \frac{1}{x}-\ln \biggl( 1+ \frac{1}{x} \biggr) - \frac{1}{2x^{2}}+\frac{1}{3x^{3}}< \sum_{j=4}^{2m} \frac{(-1)^{j}}{jx ^{j}}. $$

Using (2.12), we find that

$$\begin{aligned} &\frac{1}{x}-\ln \biggl( 1+\frac{1}{x} \biggr) - \frac{1}{2x^{2}}+\frac{1}{3x ^{3}}-a(x)+\frac{1}{4}a(x+1) \\ &\quad >\frac{1}{4x^{4}}-\frac{1}{5x^{5}}-a(x)+\frac{1}{4}a(x+1)= \frac{310x ^{4}+770x^{3}+845x^{2}+445x+92}{180x^{5}(x+1)^{5}}>0 \end{aligned}$$

and

$$\begin{aligned} &\frac{1}{x}-\ln \biggl( 1+\frac{1}{x} \biggr) - \frac{1}{2x^{2}}+\frac{1}{3x ^{3}}-b(x)+\frac{1}{4}b(x+1) \\ &\quad < \frac{1}{4x^{4}}-\frac{1}{5x^{5}}+\frac{1}{6x^{6}}-b(x)+ \frac{1}{4}b(x+1) \\ &\quad =-\frac{4380x^{5}+14{,}205x^{4}+21{,}530x^{3}+17{,}439x^{2}+7344x+1270}{540x ^{6}(x+1)^{6}}< 0. \end{aligned}$$

The proof of Lemma 2.2 is complete. □

### Remark 2.1

Using the methods from [20–22] it is possible to get estimations (based on the power series expansions) of the logarithm function that can be used, for example, in the analysis of parameterized Euler-constant function, which will be an item for further work.

### Lemma 2.3

*As* $x\to \infty $, *we have*

2.13 $$ \frac{1}{x}-\ln \biggl( 1+\frac{1}{x} \biggr) \sim C(x)+C(x+1), $$

*where* $C(x)$ *is defined by*

2.14 $$ C(x)=\sum_{j=2}^{\infty } \frac{c_{j}}{x^{j}} $$

*with the coefficients* $c_{j}$ *given by the recurrence relation*

2.15 $$\begin{aligned} c_{2}=\frac{1}{4}, \quad\quad c_{j}= \frac{(-1)^{j}}{2j}-\frac{1}{2}\sum_{k=2}^{j-1}c_{k}(-1)^{j-k} \binom{j-1}{j-k}, \quad j\geq 3. \end{aligned}$$

### Proof

In view of (2.4), we can let

2.16 $$ \frac{1}{x}-\ln \biggl( 1+\frac{1}{x} \biggr) \sim \sum_{j=2}^{\infty }\frac{c _{j}}{x^{j}}+\sum _{j=2}^{\infty }\frac{c_{j}}{(x+1)^{j}}, $$

where $c_{j}$ are real numbers to be determined. Write (2.16) as

$$ \frac{1}{x}-\ln \biggl( 1+\frac{1}{x} \biggr) \sim \sum _{j=2}^{\infty }\frac{c _{j}}{x^{j}}+\sum _{j=2}^{\infty }\frac{c_{j}}{x^{j}} \biggl( 1+ \frac{1}{x} \biggr) ^{-j}. $$

Noting that (2.7) holds, we have

2.17 $$ \sum_{j=2}^{\infty } \frac{(-1)^{j}}{j}\frac{1}{x^{j}}\sim \sum_{j=2} ^{\infty } \Biggl\{ c_{j}+\sum_{k=2}^{j}c_{k}(-1)^{j-k} \binom{j-1}{j-k} \Biggr\} \frac{1}{x ^{j}}. $$

Equating coefficients of the term $x^{-j}$ on both sides of (2.17) yields

$$ \frac{(-1)^{j}}{j}=c_{j}+\sum_{k=2}^{j}c_{k}(-1)^{j-k} \binom{j-1}{j-k}, \quad j\geq 2. $$

For $j=2$, we obtain $c_{2}=1/4$, and for $j\geq 3$ we have

$$ \frac{(-1)^{j}}{j}=2c_{j}+\sum_{k=2}^{j-1}c_{k}(-1)^{j-k} \binom{j-1}{j-k}, \quad j\geq 3. $$

We then obtain the recursive formula (2.15). The proof of Lemma 2.3 is complete. □

The first few coefficients $c_{j}$ are

$$\begin{aligned}& c_{2}=\frac{1}{4}, \quad\quad c_{3}= \frac{1}{12}, \quad\quad c_{4}=-\frac{1}{8}, \quad\quad c_{5}=- \frac{1}{10},\quad\quad c_{6}= \frac{1}{4}, \\& c_{7}= \frac{17}{56},\quad\quad c_{8}=- \frac{17}{16},\quad\quad c_{9}=-\frac{31}{18}. \end{aligned}$$

## Main results

For any positive integer $m\geq 2$, Theorem 3.1 gives the asymptotic expansion of $\gamma ( 1/m ) -\gamma_{n} ( 1/m ) $ as $n\to \infty $.

### Theorem 3.1

*For any positive integer* $m\geq 2$, *we have*

3.1 $$\begin{aligned} \gamma \biggl( \frac{1}{m} \biggr) -\gamma_{n} \biggl( \frac{1}{m} \biggr) \sim \sum_{k=n+1}^{\infty } \sum_{j=2}^{m-1}\frac{(-1)^{j}}{j\cdot m ^{k-1}} \frac{1}{k^{j}}+\frac{A(n+1)}{m^{n}},\quad n\to \infty , \end{aligned}$$

*where* $A(x)$ *is given in* (2.2). *Namely*,

3.2 $$\begin{aligned} &\gamma \biggl( \frac{1}{m} \biggr) -\gamma_{n} \biggl( \frac{1}{m} \biggr) \\ & \quad \sim \sum_{k=n+1}^{\infty } \sum _{j=2}^{m-1}\frac{(-1)^{j}}{j\cdot m ^{k-1}} \frac{1}{k^{j}} \\ &\quad \quad {}+\frac{(-1)^{m}}{m^{n}} \biggl\{ \frac{1}{(m-1)(n+1)^{m}}- \frac{2m ^{2}}{(m+1)(m-1)^{2}(n+1)^{m+1}} \\ &\quad \quad {}+\frac{m^{2}(m^{2}+8m+3)}{2(m+2)(m-1)^{3}(n+1)^{m+2}}-\frac{(m+1)(m ^{3}+12m^{2}+51m+8)m^{2}}{6(m-1)^{4}(m+3)(n+1)^{m+3}} \\ & \quad \quad {}+ \frac{m^{2}(m^{6}+25m^{5}+216m^{4}+866m^{3}+1241m^{2}+501m+30)}{24(m-1)^{5}(m+4)(n+1)^{m+4}}- \cdots \biggr\} . \end{aligned}$$

### Proof

Write (2.1) as

3.3 $$ \frac{1}{k}-\ln \biggl( 1+\frac{1}{k} \biggr) -\sum _{j=2}^{m-1} \frac{(-1)^{j}}{j} \frac{1}{k^{j}}= A_{N}(k)-\frac{1}{m}A_{N}(k+1)+O \biggl( \frac{1}{k^{N+1}} \biggr) , $$

where

3.4 $$ A_{N}(k)=\sum_{j=m}^{N} \frac{a_{j}}{k^{j}} $$

with the coefficients $a_{j}$ given by the recurrence relation (2.3). From (3.3), we have

3.5 $$\begin{aligned} &\frac{1}{m^{k-1}} \biggl( \frac{1}{k}-\ln \biggl( 1+ \frac{1}{k} \biggr) \biggr) -\frac{1}{m ^{k-1}}\sum _{j=2}^{m-1}\frac{(-1)^{j}}{j}\frac{1}{k^{j}} \\ & \quad = \frac{A_{N}(k)}{m^{k-1}}-\frac{A_{N}(k+1)}{m^{k}}+O \biggl( \frac{1}{m ^{k-1}k^{N+1}} \biggr) . \end{aligned}$$

Adding (3.5) from $k=n+1$ to $k=\infty $, we have

$$\begin{aligned} &\gamma \biggl( \frac{1}{m} \biggr) -\gamma_{n} \biggl( \frac{1}{m} \biggr) - \sum_{k=n+1}^{\infty } \sum_{j=2}^{m-1}\frac{(-1)^{j}}{j\cdot m^{k-1}} \frac{1}{k ^{j}}=\frac{1}{m^{n}} \biggl\{ A_{N}(n+1)+O \biggl( \frac{1}{(n+1)^{N+1}} \biggr) \biggr\} , \end{aligned}$$

which can be written as (3.1). The proof of Theorem 3.1 is complete. □

### Remark 3.1

For $m=2$ in (3.2), we obtain (1.15). For $m=3$ in (3.2), we find

3.6 $$\begin{aligned} &\gamma \biggl( \frac{1}{3} \biggr) -\gamma_{n} \biggl( \frac{1}{3} \biggr) \\ &\quad \sim \sum_{k=n+1}^{\infty } \frac{1}{2k^{2} 3^{k-1}} \\ &\quad\quad {}+\frac{1}{3^{n}(n+1)^{3}} \biggl\{ -\frac{1}{2}+ \frac{9}{8(n+1)}- \frac{81}{20(n+1)^{2}}+\frac{37}{2(n+1)^{3}}- \frac{5661}{56(n+1)^{4}}+ \cdots \biggr\} . \end{aligned}$$

For $m=4$ in (3.2), we find

3.7 $$\begin{aligned} &\gamma \biggl( \frac{1}{4} \biggr) -\gamma_{n} \biggl( \frac{1}{4} \biggr) \\ &\quad \sim \sum_{k=n+1}^{\infty } \biggl( \frac{1}{2}-\frac{1}{3k} \biggr) \frac{1}{k ^{2}4^{k-1}} \\ &\quad\quad {}+\frac{1}{4^{n}(n+1)^{4}} \biggl\{ \frac{1}{3}- \frac{32}{45(n+1)}+ \frac{68}{27(n+1)^{2}}-\frac{2080}{189(n+1)^{3}} \\ &\quad\quad {}+ \frac{9017}{162(n+1)^{4}}-\cdots \biggr\} . \end{aligned}$$

Formula (3.7) motivated us to establish Theorem 3.2.

### Theorem 3.2

*For* $n\in \mathbb{N}$,

3.8 $$\begin{aligned} &\sum_{k=n+1}^{\infty } \biggl( \frac{1}{2}- \frac{1}{3k} \biggr) \frac{1}{k ^{2}4^{k-1}}+ \frac{1}{4^{n}} \biggl\{ \frac{1}{3(n+1)^{4}}- \frac{32}{45(n+1)^{5}} \biggr\} \\ &\quad< \gamma \biggl( \frac{1}{4} \biggr) -\gamma _{n} \biggl( \frac{1}{4} \biggr) \\ &\quad < \sum_{k=n+1}^{\infty } \biggl( \frac{1}{2}-\frac{1}{3k} \biggr) \frac{1}{k ^{2}4^{k-1}}+ \frac{1}{4^{n}} \biggl\{ \frac{1}{3(n+1)^{4}}- \frac{32}{45(n+1)^{5}}+ \frac{68}{27(n+1)^{6}} \biggr\} . \end{aligned}$$

### Proof

From the double inequality (2.11), we have

3.9 $$ \begin{aligned}[b] \frac{a(k)}{4^{k-1}}-\frac{a(k+1)}{4^{k}}&< \frac{1}{4^{k-1}} \biggl( \frac{1}{k}- \ln \biggl( 1+\frac{1}{k} \biggr) \biggr) - \frac{1}{4^{k-1}} \biggl( \frac{1}{2k ^{2}}- \frac{1}{3k^{3}} \biggr) \\ &< \frac{b(k)}{4^{k-1}}- \frac{b(k+1)}{4^{k}},\end{aligned} $$

where $a(x)$ and $b(x)$ are given in (2.10). Adding inequalities (3.9) from $k=n+1$ to $k=\infty $, we have

$$ \frac{a(n+1)}{4^{n}}< \sum_{k=n+1}^{\infty } \frac{1}{4^{k-1}} \biggl( \frac{1}{k}- \ln \biggl( 1+\frac{1}{k} \biggr) \biggr) -\sum_{k=n+1}^{\infty } \frac{1}{4^{k-1}} \biggl( \frac{1}{2k^{2}}-\frac{1}{3k^{3}} \biggr) < \frac{b(n+1)}{4^{n}}, $$

which can be written as (3.8). The proof of Theorem 3.2 is complete. □

### Remark 3.2

Inequality (3.8) can be further refined by inserting additional terms on both sides of the inequality. For example, for $n\in \mathbb{N}$, we have

3.10 $$\begin{aligned} &\sum_{k=n+1}^{\infty } \biggl( \frac{1}{2}- \frac{1}{3k} \biggr) \frac{1}{k ^{2}4^{k-1}}+ \frac{1}{4^{n}} \biggl\{ \frac{1}{3(n+1)^{4}}- \frac{32}{45(n+1)^{5}}+ \frac{68}{27(n+1)^{6}}- \frac{2080}{189(n+1)^{7}} \biggr\} \\ &\quad < \gamma \biggl( \frac{1}{4} \biggr) -\gamma_{n} \biggl( \frac{1}{4} \biggr) \\ &\quad < \sum_{k=n+1}^{\infty } \biggl( \frac{1}{2}-\frac{1}{3k} \biggr) \frac{1}{k ^{2}4^{k-1}} \\ & \qquad {}+\frac{1}{4^{n}} \biggl\{ \frac{1}{3(n+1)^{4}}-\frac{32}{45(n+1)^{5}}+ \frac{68}{27(n+1)^{6}}-\frac{2080}{189(n+1)^{7}}+ \frac{9017}{162(n+1)^{8}} \biggr\} . \end{aligned}$$

### Remark 3.3

Following the same method as the one used in the proof of Theorem 3.2, we can prove the following inequality:

3.11 $$\begin{aligned} &\sum_{k=n+1}^{\infty } \frac{1}{2k^{2}3^{k-1}}+ \frac{1}{3^{n}} \biggl\{ -\frac{1}{2(n+1)^{3}}+ \frac{9}{8(n+1)^{4}}- \frac{81}{20(n+1)^{5}}+ \frac{37}{2(n+1)^{6}}-\frac{5661}{56(n+1)^{7}} \biggr\} \\ &\quad < \gamma \biggl( \frac{1}{3} \biggr) -\gamma_{n} \biggl( \frac{1}{3} \biggr) \\ &\quad < \sum_{k=n+1}^{\infty }\frac{1}{2k^{2} 3^{k-1}}+ \frac{1}{3^{n}} \biggl\{ -\frac{1}{2(n+1)^{3}}+\frac{9}{8(n+1)^{4}}- \frac{81}{20(n+1)^{5}}+\frac{37}{2(n+1)^{6}} \biggr\} \end{aligned}$$

for $n\in \mathbb{N}$. We omit the proof.

In view of (1.14), (3.11), (3.8), and (3.10), we pose the following conjecture.

### Conjecture 3.1

For any positive integer $m\geq 2$, we have

3.12 $$\begin{aligned} \begin{aligned}[b] \frac{(-1)^{m}}{m^{n}}\sum_{j=m}^{2N} \frac{a_{j}}{(n+1)^{j}} &< (-1)^{m} \Biggl\{ \gamma \biggl( \frac{1}{m} \biggr) -\gamma_{n} \biggl( \frac{1}{m} \biggr) - \sum_{k=n+1}^{\infty }\sum _{j=2}^{m-1}\frac{(-1)^{j}}{j\cdot m^{k-1}k ^{j}} \Biggr\} \\ &< \frac{(-1)^{m}}{m^{n}}\sum_{j=m}^{2N+1} \frac{a_{j}}{(n+1)^{j}}, \end{aligned} \end{aligned}$$

with the coefficients $a_{j}$ given in (2.3).

By using the Maple software, we find, as $n\to \infty $,

3.13 $$\begin{aligned}& \gamma \biggl( \frac{1}{2} \biggr) -\gamma_{n} \biggl( \frac{1}{2} \biggr) \sim \frac{1}{2^{n}(n+1)^{2}} \biggl(1+ \frac{-\frac{8}{3}}{n+ \frac{85}{16}}+\frac{-\frac{2689}{160}}{ (n+\frac{807{,}797}{129{,}072} )^{3}}+\cdots \biggr), \end{aligned}$$

3.14 $$\begin{aligned}& \begin{aligned}[b] & \gamma \biggl( \frac{1}{3} \biggr) - \gamma_{n} \biggl( \frac{1}{3} \biggr) \\ & \quad \sim \sum_{k=n+1}^{\infty } \frac{1}{2k^{2} 3^{k-1}}+ \frac{1}{3^{n}(n+1)^{3}} \biggl(-\frac{1}{2}+ \frac{\frac{9}{8}}{n+ \frac{23}{5}}+ \frac{\frac{98}{25}}{ (n+\frac{140{,}843}{27{,}440} )^{3}}+\cdots \biggr) \end{aligned} \end{aligned}$$

and

3.15 $$\begin{aligned} \gamma \biggl( \frac{1}{4} \biggr) -\gamma_{n} \biggl( \frac{1}{4} \biggr) & \sim \sum_{k=n+1}^{\infty } \biggl( \frac{1}{2}-\frac{1}{3k} \biggr) \frac{1}{k ^{2}4^{k-1}} \\ &\quad {}+\frac{1}{4^{n}(n+1)^{4}} \biggl\{ \frac{1}{3}+\frac{- \frac{32}{45}}{n+\frac{109}{24}}+ \frac{-\frac{2365}{1134}}{ (n+ \frac{825{,}361}{170{,}280} )^{3}}+\cdots \biggr\} . \end{aligned}$$

From a computational viewpoint, formulas (3.13), (3.14), and (3.15) improve formulas (1.15), (3.6), and (3.7), respectively.

For any positive integer $m\geq 2$, we here provide a pair of recurrence relations for determining the constants $p_{\ell }\equiv p_{\ell }(m)$ and $q_{\ell }\equiv q_{\ell }(m)$ (see Remark 3.4) such that

3.16 $$\begin{aligned} \begin{aligned}[b]& \gamma \biggl( \frac{1}{m} \biggr) - \gamma_{n} \biggl( \frac{1}{m} \biggr) \\ & \quad \sim \sum_{k=n+1}^{\infty } \sum _{j=2}^{m-1}\frac{(-1)^{j}}{j\cdot m ^{k-1}} \frac{1}{k^{j}}+ \frac{1}{m^{n}(n+1)^{m}} \Biggl( a_{m}+\sum_{ \ell =1}^{\infty } \frac{p_{\ell }}{(n+q_{\ell })^{2\ell -1}} \Biggr) \end{aligned} \end{aligned}$$

as $n\to \infty $. This develops formulas (3.13), (3.14), and (3.15) to produce a general result given by Theorem 3.3.

### Theorem 3.3

*For any positive integer* $m\geq 2$, *we have*

3.17 $$\begin{aligned} \gamma \biggl( \frac{1}{m} \biggr) -\gamma_{n-1} \biggl( \frac{1}{m} \biggr) \sim \sum_{k=n}^{\infty } \sum_{j=2}^{m-1} \frac{(-1)^{j}}{j\cdot m^{k-1}} \frac{1}{k^{j}}+\frac{1}{m^{n-1}n^{m}} \Biggl( a_{m}+\sum _{\ell =1}^{\infty }\frac{\lambda_{\ell }}{(n+\mu_{ \ell })^{2\ell -1}} \Biggr) \end{aligned}$$

*as* $n\to \infty $, *where* $\lambda_{\ell }\equiv \lambda_{\ell }(m)$ *and* $\mu_{\ell }\equiv \mu_{\ell }(m)$ *are given by a pair of recurrence relations*

3.18 $$ \lambda_{\ell }=a_{m+2\ell -1}-\sum _{k=1}^{\ell -1}\lambda_{k}\mu_{k} ^{2\ell -2k}\binom{2\ell -2}{2\ell -2k}, \quad \ell \geq 2, $$

*and*

3.19 $$\begin{aligned} \mu_{\ell }=-\frac{1}{(2\ell -1)\lambda_{\ell }} \Biggl\{ a_{m+2\ell }+ \sum_{k=1}^{\ell -1} \lambda_{k}\mu_{k}^{2\ell -2k+1}\binom{2\ell -1}{2 \ell -2k+1} \Biggr\} , \quad \ell \geq 2, \end{aligned}$$

*with*

$$\begin{aligned} \lambda_{1}=a_{m+1}=\frac{(-1)^{m+1}2m^{2}}{(m+1)(m-1)^{2}}\quad \textit{and}\quad \mu_{1}=-\frac{a_{m+2}}{\lambda_{1}}=\frac{(m+1)(m ^{2}+8m+3)}{4(m+2)(m-1)}. \end{aligned}$$

*Here* $a_{j}$ *are given in* (2.3).

### Proof

In view of (3.13), (3.14), and (3.15), we let

$$\begin{aligned} \gamma \biggl( \frac{1}{m} \biggr) -\gamma_{n-1} \biggl( \frac{1}{m} \biggr) \sim \sum_{k=n}^{\infty } \sum_{j=2}^{m-1} \frac{(-1)^{j}}{j\cdot m^{k-1}} \frac{1}{k^{j}}+\frac{1}{m^{n-1}n^{m}} \Biggl( a_{m}+\sum _{\ell =1}^{\infty }\frac{\lambda_{\ell }}{(n+\mu_{ \ell })^{2\ell -1}} \Biggr) , \end{aligned}$$

where $\lambda_{\ell }$ and $\mu_{\ell }$ are real numbers to be determined. This can be written as

3.20 $$\begin{aligned} \begin{aligned}[b] & m^{n-1}n^{m} \Biggl\{ \gamma \biggl( \frac{1}{m} \biggr) -\gamma_{n-1} \biggl( \frac{1}{m} \biggr) - \sum_{k=n}^{\infty } \sum_{j=2}^{m-1}\frac{(-1)^{j}}{j\cdot m^{k-1}} \frac{1}{k ^{j}} \Biggr\} \\ & \quad \sim a_{m}+\sum_{j=1}^{\infty } \frac{\lambda_{j}}{n^{2j-1}} \biggl( 1+\frac{\mu_{j}}{n} \biggr) ^{-2j+1}. \end{aligned} \end{aligned}$$

Direct computation yields

$$\begin{aligned} \sum_{j=1}^{\infty }\frac{\lambda_{j}}{n^{2j-1}} \biggl( 1+ \frac{\mu_{j}}{n} \biggr) ^{-2j+1} &=\sum _{j=1}^{\infty }\frac{\lambda _{j}}{n^{2j-1}}\sum _{k=0}^{\infty }\binom{-2j+1}{k}\frac{\mu_{j}^{k}}{n ^{k}} \\ &= \sum_{j=1}^{\infty }\frac{\lambda_{j}}{n^{2j-1}}\sum _{k=0}^{ \infty }(-1)^{k} \binom{k+2j-2}{k}\frac{\mu_{j}^{k}}{n^{k}} \\ &= \sum_{j=1}^{\infty }\sum _{k=0}^{j-1}\lambda_{k+1}\mu_{k+1}^{j-k-1}(-1)^{j-k-1} \binom{j+k-1}{j-k-1}\frac{1}{n^{j+k}}, \end{aligned}$$

which can be written as

3.21 $$\begin{aligned} \sum_{j=1}^{\infty } \frac{\lambda_{j}}{n^{2j-1}} \biggl( 1+ \frac{\mu_{j}}{n} \biggr) ^{-2j+1} \sim \sum_{j=1}^{\infty } \Biggl\{ \sum _{k=1} ^{\lfloor \frac{j+2}{2}\rfloor }\lambda_{k}\mu_{k}^{j-2k+1}(-1)^{j-1} \binom{j-1}{j-2k+1} \Biggr\} \frac{1}{n^{j}}. \end{aligned}$$

Substituting (3.21) into (3.20) we have

3.22 $$\begin{aligned} &m^{n-1}n^{m} \Biggl\{ \gamma \biggl( \frac{1}{m} \biggr) -\gamma_{n-1} \biggl( \frac{1}{m} \biggr) - \sum _{k=n}^{\infty }\sum_{j=2}^{m-1} \frac{(-1)^{j}}{j\cdot m^{k-1}}\frac{1}{k ^{j}} \Biggr\} \\ & \quad \sim a_{m}+\sum_{j=1}^{\infty } \Biggl\{ \sum_{k=1}^{\lfloor \frac{j+2}{2}\rfloor } \lambda_{k}\mu_{k}^{j-2k+1}(-1)^{j-1} \binom{j-1}{j-2k+1} \Biggr\} \frac{1}{n^{j}}. \end{aligned}$$

On the other hand, it follows from (3.1) that

3.23 $$\begin{aligned} m^{n-1}n^{m} \Biggl\{ \gamma \biggl( \frac{1}{m} \biggr) -\gamma_{n-1} \biggl( \frac{1}{m} \biggr) - \sum_{k=n}^{\infty }\sum _{j=2}^{m-1}\frac{(-1)^{j}}{j\cdot m^{k-1}}\frac{1}{k ^{j}} \Biggr\} \sim \sum_{j=0}^{\infty } \frac{a_{m+j}}{n^{j}}. \end{aligned}$$

Equating coefficients of the term $n^{-j}$ on the right-hand sides of (3.22) and (3.23), we obtain

3.24 $$ a_{m+j}=\sum_{k=1}^{\lfloor \frac{j+2}{2}\rfloor } \lambda_{k}\mu_{k} ^{j-2k+1}(-1)^{j-1} \binom{j-1}{j-2k+1}, \quad j\in \mathbb{N}. $$

Setting $j=2\ell -1$ and $j=2\ell $ in (3.24), respectively, yields

3.25 $$ a_{m+2\ell -1}=\sum_{k=1}^{\ell } \lambda_{k}\mu_{k}^{2\ell -2k}\binom{2 \ell -2}{2\ell -2k} $$

and

3.26 $$\begin{aligned} a_{m+2\ell } &=-\sum_{k=1}^{\ell +1} \lambda_{k}\mu_{k}^{2\ell -2k+1}\binom{2 \ell -1}{2\ell -2k+1} \\ &=-\sum_{k=1}^{\ell }\lambda_{k} \mu_{k}^{2\ell -2k+1}\binom{2\ell -1}{2 \ell -2k+1}-\lambda_{\ell +1} \mu_{\ell +1}^{-1}\binom{2\ell -1}{-1} \\ &=-\sum_{k=1}^{\ell }\lambda_{k} \mu_{k}^{2\ell -2k+1}\binom{2\ell -1}{2 \ell -2k+1}. \end{aligned}$$

For $\ell =1$, from (3.25) and (3.26) we obtain

$$\begin{aligned} \lambda_{1}=a_{m+1}=\frac{(-1)^{m+1}2m^{2}}{(m+1)(m-1)^{2}}\quad \text{and}\quad \mu_{1}=-\frac{a_{m+2}}{\lambda_{1}}=\frac{(m+1)(m ^{2}+8m+3)}{4(m+2)(m-1)}, \end{aligned}$$

and for $\ell \geq 2$ we have

$$ a_{m+2\ell -1}=\sum_{k=1}^{\ell -1} \lambda_{k}\mu_{k}^{2\ell -2k}\binom{2 \ell -2}{2\ell -2k}+ \lambda_{\ell } $$

and

$$\begin{aligned} a_{m+2\ell } &=-\sum_{k=1}^{\ell -1} \lambda_{k}\mu_{k}^{2\ell -2k+1}\binom{2 \ell -1}{2\ell -2k+1}-(2\ell -1) \lambda_{\ell }\mu_{\ell }. \end{aligned}$$

We then obtain the recurrence relations (3.18) and (3.19). The proof of Theorem 3.3 is complete. □

Here we give explicit numerical values of some first terms of $\lambda_{\ell }$ and $\mu_{\ell }$ by using formulas (3.18) and (3.19). This shows how easily we can determine the constants $a_{\ell }$ and $b_{\ell }$ in (3.17).

$$\begin{aligned}& \lambda_{1}=a_{m+1}=\frac{(-1)^{m+1}2m^{2}}{(m+1)(m-1)^{2}}, \\& \mu_{1}=-\frac{a_{m+2}}{\lambda_{1}}= \frac{(m+1)(m^{2}+8m+3)}{4(m+2)(m-1)}, \\& \begin{aligned} \lambda_{2} &=a_{m+3}-\lambda_{1} \mu_{1}^{2} \\ &=\frac{(-1)^{m+1}m^{2}(m+1)(m^{3}+12m^{2}+51m+8)}{6(m-1)^{4}(m+3)} \\ &\quad {}-\frac{(-1)^{m+1}2m^{2}}{(m+1)(m-1)^{2}} \biggl( \frac{(m+1)(m ^{2}+8m+3)}{4(m+2)(m-1)} \biggr) ^{2} \\ &=(-1)^{m+1} \frac{m^{2}(m+1)(m^{5}+7m^{4}+58m^{3}+266m^{2}+485m+47)}{24(m-1)^{4}(m+3)(m+2)^{2}}, \end{aligned} \\& \begin{aligned} \mu_{2} &=-\frac{a_{m+4}+\lambda_{1}\mu_{1}^{3}}{3\lambda_{2}} \\ &=-\frac{\frac{(-1)^{m}m^{2}(m^{6}+25m^{5}+216m^{4}+866m^{3}+1241m ^{2}+501m+30)}{24(m-1)^{5}(m+4)}+\lambda_{1}\mu_{1}^{3}}{3\lambda_{2}} \\ &=\bigl((m+3) \bigl(m^{9}+34m^{8}+450m^{7}+3634m^{6}+17{,}584m^{5}+48{,}642m ^{4}+71{,}302m^{3} \\ &\quad{} +50{,}926m^{2}+14{,}151m+636\bigr)\bigr) /\bigl(12(m+2) (m+4) \bigl(m^{5}+7m^{4}+58m ^{3}+266m^{2} \\ &\quad{}+485m+47\bigr) \bigl(m^{2}-1\bigr)\bigr). \end{aligned} \end{aligned}$$

### Remark 3.4

The constants $p_{\ell }$ and $q_{\ell }$ in (3.16) are given by

$$ p_{\ell }:=\lambda_{\ell }\quad \text{and}\quad q_{\ell }:=1+\mu_{ \ell }. $$

Setting $m=2, 3$, and 4 in (3.16), respectively, yields (3.13), (3.14), and (3.15).

Noting that $\ln \frac{4}{\pi } = \gamma (-1)$ holds, Theorem 3.4 presents the asymptotic expansion for $\ln \frac{4}{\pi }$.

### Theorem 3.4

*As* $n\to \infty $, *we have*

3.27 $$ \gamma ( -1 ) -\gamma_{n} ( -1 ) \sim (-1)^{n}C(n+1), $$

*where* $C(x)$ *is given in* (2.14). *Namely*,

3.28 $$\begin{aligned} \begin{aligned}[b] &\gamma ( -1 ) -\gamma_{n} ( -1 ) \\ &\quad \sim (-1)^{n} \biggl\{ \frac{1}{4(n+1)^{2}}+\frac{1}{12(n+1)^{3}}- \frac{1}{8(n+1)^{4}}- \frac{1}{10(n+1)^{5}}+\cdots \biggr\} .\end{aligned} \end{aligned}$$

### Proof

Write (2.13) as

3.29 $$ \frac{1}{k}-\ln \biggl( 1+\frac{1}{k} \biggr) =C_{N}(k)+C_{N}(k+1)+O \biggl( \frac{1}{k ^{N+1}} \biggr) , $$

where

3.30 $$ C_{N}(x)=\sum_{j=2}^{N} \frac{c_{j}}{x^{j}} $$

with the coefficients $c_{j}$ given by the recurrence relation (2.15).

From (3.29), we have

3.31 $$ (-1)^{k-1} \biggl( \frac{1}{k}-\ln \biggl( 1+ \frac{1}{k} \biggr) \biggr) =(-1)^{k-1}C _{N}(k)-(-1)^{k}C_{N}(k+1)+O \biggl( \frac{(-1)^{k-1}}{k^{N+1}} \biggr) . $$

Adding (3.31) from $k=n+1$ to $k=\infty $, we have

3.32 $$\begin{aligned} \gamma ( -1 ) -\gamma_{n} ( -1 ) &=\sum_{k=n+1}^{ \infty }(-1)^{k-1} \biggl( \frac{1}{k}-\ln \biggl( 1+\frac{1}{k} \biggr) \biggr) \\ &=(-1)^{n}C_{N}(n+1)+O \biggl( \frac{1}{(n+1)^{N+1}} \biggr) , \end{aligned}$$

which can be written as (3.27). The proof of Theorem 3.4 is complete. □

### Remark 3.5

We see from (3.28) that the alternating Euler constant $\ln \frac{4}{\pi }$ has the following expansion:

3.33 $$\begin{aligned} \ln \frac{4}{\pi } &\sim \sum_{k=1}^{n}(-1)^{k-1} \biggl( \frac{1}{k}- \ln \frac{k+1}{k} \biggr) \\ &\quad {}+(-1)^{n} \biggl\{ \frac{1}{4(n+1)^{2}}+\frac{1}{12(n+1)^{3}}- \frac{1}{8(n+1)^{4}}-\frac{1}{10(n+1)^{5}}+\cdots \biggr\} . \end{aligned}$$

## Conclusions

In this paper, we give asymptotic expansions related to the generalized Somos quadratic recurrence constant (Theorems 3.1 and 3.3). We present the inequalities for $\gamma ( \frac{1}{4} ) - \gamma_{n} ( \frac{1}{4} ) $ and $\gamma ( \frac{1}{3} ) - \gamma_{n} ( \frac{1}{3} ) $ (see (3.8), (3.10), and (3.11)). The expansion of the alternating Euler constant $\ln \frac{4}{\pi }$ is also obtained (see (3.33)).
